# Staged Repair Using Modified Bishop-Koop Procedure in Complicated Congenital Colonic Atresia in a Neonate

**DOI:** 10.7759/cureus.18149

**Published:** 2021-09-21

**Authors:** Sultan A Neazy, Hisham A Basamh, Jamal Kamal, Rana M Alghamdi, Alanoud S Bin Suayb

**Affiliations:** 1 Pediatric Surgery, College of Medicine, King Saud bin Abdulaziz University for Health Sciences, King Abdullah International Medical Research Centre, King Abdulaziz Medical City, National Guard Health Affairs, Jeddah, SAU; 2 Pediatric Surgery, King Abdulaziz University Hospital, Jeddah, SAU; 3 Surgery, College of Medicine, Imam Mohammed Ibn Saud Islamic University, Riyadh, SAU

**Keywords:** stoma closure, contrast enema, emergency stoma, bishop-koop procedure, congenital colonic atresia

## Abstract

Colonic atresia (CA) is the rarest type of intestinal atresia and is defined as an obstruction in the large intestinal lumen. This is a rare case presentation of a four-day-old full-term female patient presented with signs and symptoms of intestinal obstruction. Investigation revealed that she had complicated CA located in the splenic flexure. Laparotomy and colostomy were done on the patient. About two months later, she was admitted for stoma closure, which was converted to modified Bishop-Koop stoma. Lastly, the patient underwent a successful stoma closure. Upon one month of follow-up, the patient's condition has markedly improved and the wound healed well without any complications.

## Introduction

Colonic atresia (CA) is defined as a complete obstruction in the lumen of the large intestine. The percentage of CA ranges from 1.8% to 15% of all intestinal atresia types, which makes the colon the least common site for atresia [1]. Regarding gender, CA is more common in males compared to females [2]. In 1673, Binniger was the first physician to report a case about CA [3]. The most common site for CA is the sigmoid colon followed by splenic flexure [4]. A study done in Turkey concluded that CA patients with early diagnosis and surgical intervention (less than 72 hours) had a low mortality rate [2]. Since CA is a rare disease, there is insufficient information regarding its management and prognosis. Moreover, a good prognosis depends on proper diagnosis and management. Hence, our aim is to present a case about CA including the diagnostic approach and the outcomes of using the modified Bishop-Koop procedure in the management.

## Case presentation

A four-day-old full-term female baby was born through cesarean section due to umbilical coiling around the neck and oligohydramnios. The patient also did not pass any meconium after delivery. At the age of three days, the patient was discharged from the private hospital against medical advice (AMA) without any investigations due to financial issues.

**Figure 1:**
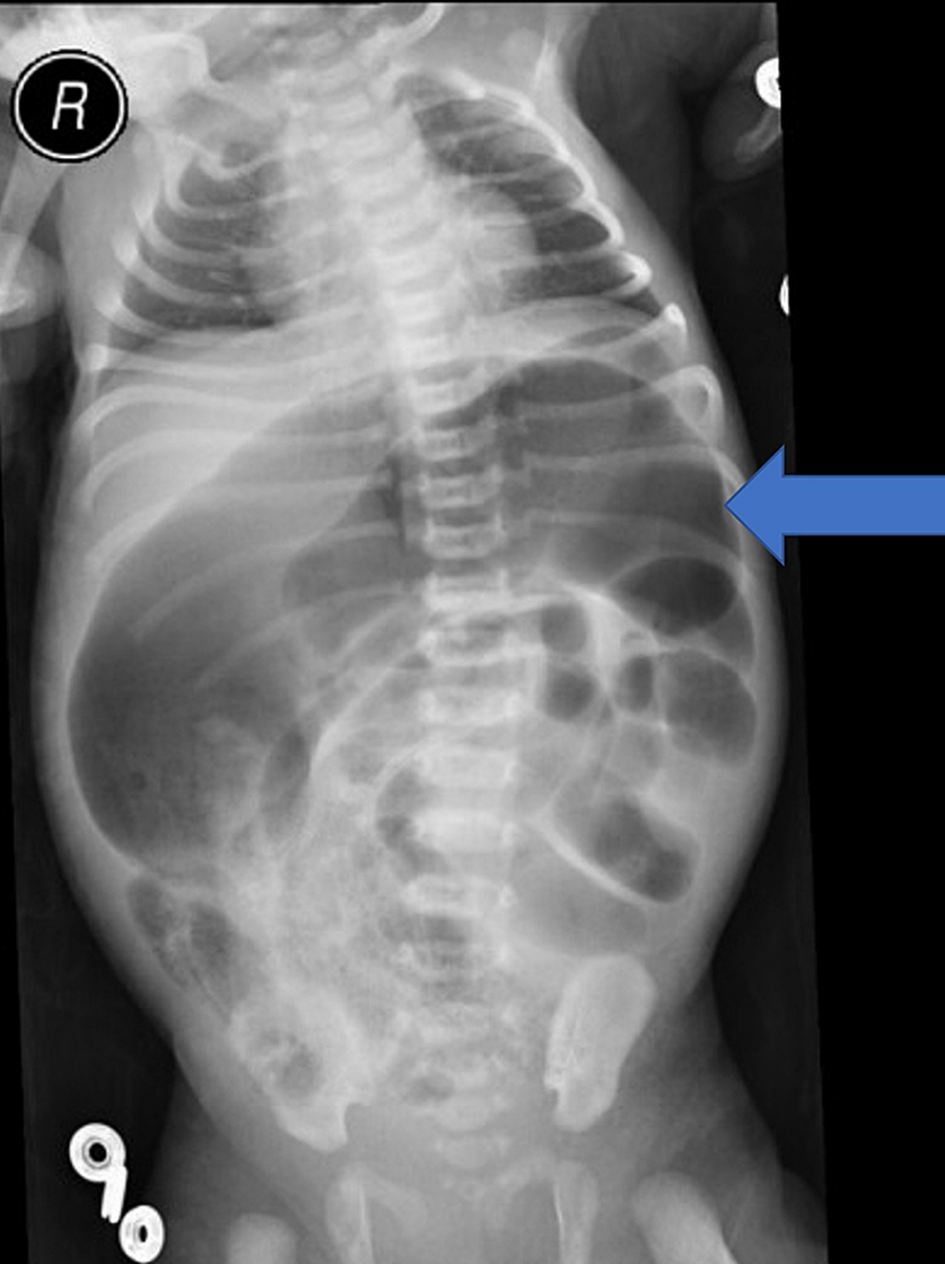
Plain abdominal x-ray showing hugely dilated bowel loops (more likely colon) and air-fluid levels with no gas distally at the rectum. Arrow: dilated left-sided bowel loops, R: right.

The baby was presented to our emergency department (ER) complaining of severe constipation and abdominal distention increasing with time associated with a high-grade fever. These symptoms progressed into vomiting that started initially with milk then turned into greenish vomiting. There was no change in urine smell or color, no decrease in activity, no cough, no runny nose, and no abnormal movement. The patient had high blood pressure (104/64), high heart rate (170), high temperature (39.5°C), high respiratory rate (70), and normal oxygen saturation (100%). Upon physical examination, the baby was active and alert with open and flat fontanels. However, she looked slightly jaundiced and dehydrated. Abdominal examination showed a tense distended abdomen with hyper-resonant percussion and positive bowel sounds. Perineum examination showed a normal female phenotype. Per-rectal examination revealed a normally located patent anal opening with an empty rectum. However, there was no stool coming out during the per-rectal examination. Lab investigations were all normal except for high total bilirubin (186 µmol/L) and C-reactive protein (CRP) (6.92). A plain abdominal x-ray showed hugely dilated left-sided bowel loops and air-fluid levels with no gas distally at the rectum (Figure 1).

**Figure 2:**
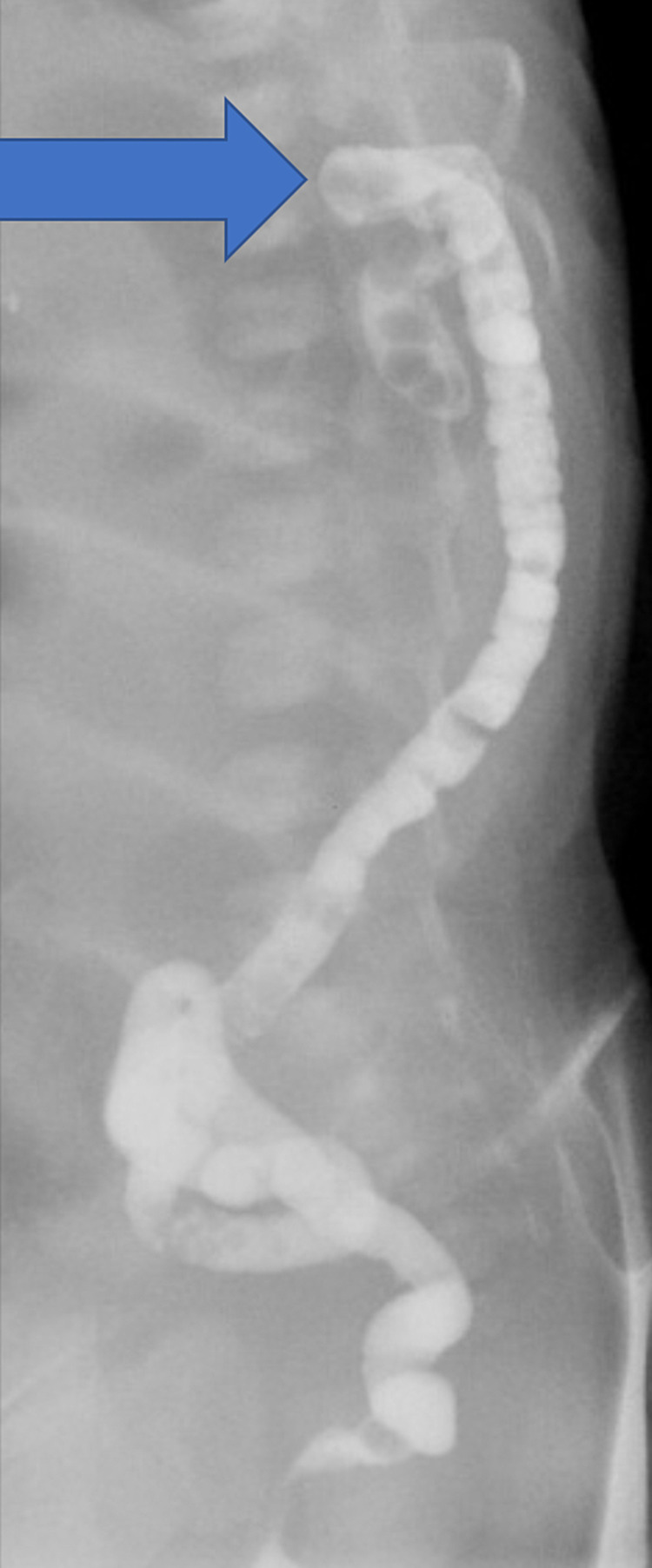
Contrast (gastrografin) enema showing that the caliber of the rectum and sigmoid portions of the colon was narrow and hypoplastic with a blind end appearance on the splenic flexure. Arrow: the blind end at the splenic flexure.

Contrast (gastrografin) enema showed that the caliber of the rectum and sigmoid portions of the colon was smaller than normal and hypoplastic. Moreover, the cutoff of the contrast was documented at the level of splenic flexure with a blind end appearance. The baby was diagnosed with CA at the splenic flexure (Figure 2).

**Figure 3:**
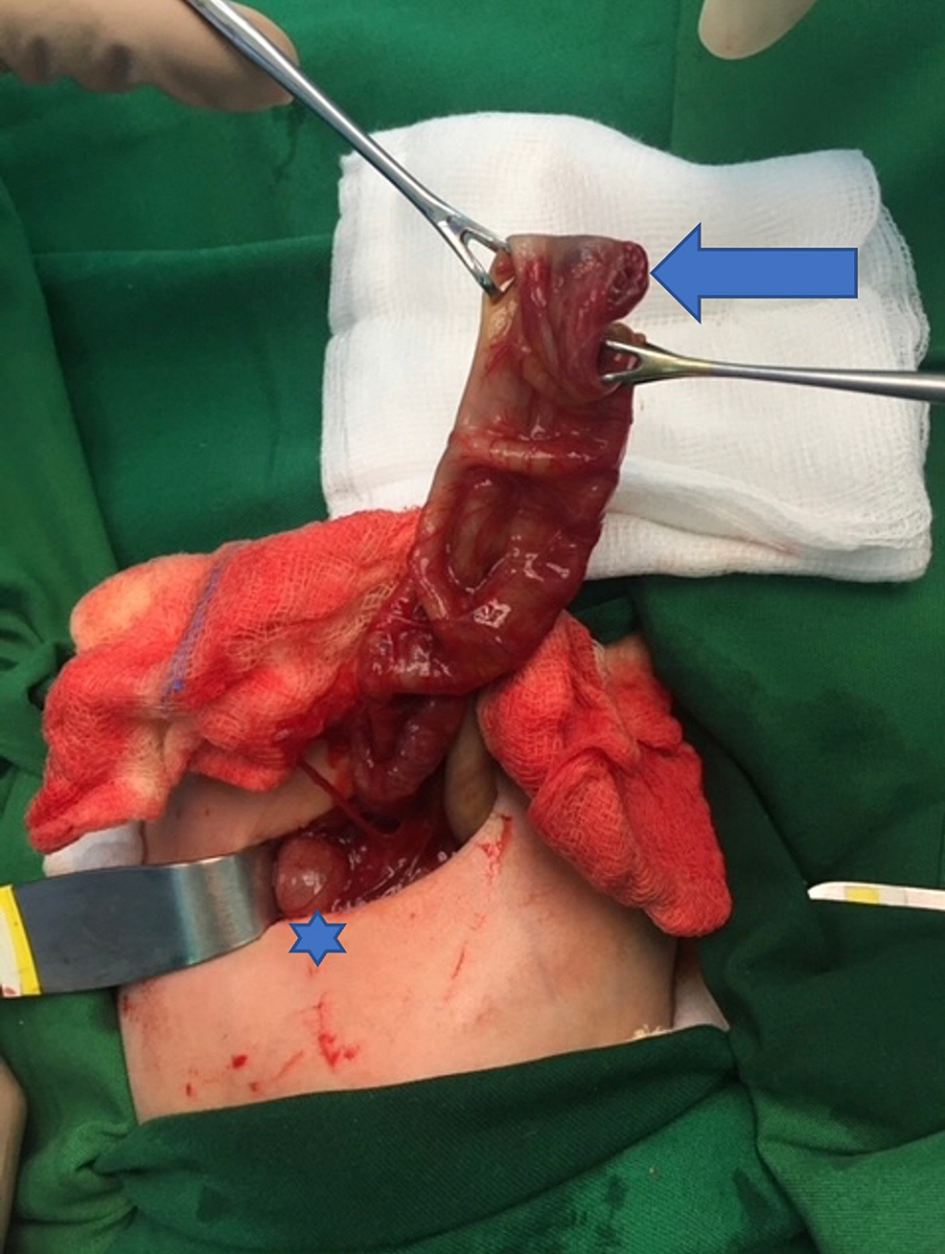
Interruption in the gastrointestinal tract with the proximal blind end in the large colon at the splenic flexure and atretic distal colonic end. Arrow: proximal blind end in the large colon at the splenic flexure, Star: the distal atretic end with a small-caliber colon.

The baby was managed initially by intravenous (IV) fluid and antibiotics (metronidazole and cefotaxime). A nasogastric (NG) tube was also inserted into the patient. After that, she was kept NPO (Nill Per Oral) and was sent to the operation room (OR). Laparotomy was performed through a right transverse supraumbilical incision. During the operation, the dilated transverse colon was followed until we reached an atretic end at the splenic flexure (Figure 3).

After that, it was brought out as a proximal stoma. The distal small colon was atretic and exteriorized as a mucous fistula. Moreover, a rectal biopsy was taken for histopathological examination and revealed normal ganglion cells, which ruled out Hirschsprung’s disease. After the operation, the patient was vitally stable, afebrile, tolerated the feeding well and the stoma was functioning. In addition, the feeding was increased gradually on the fourth day post-operation until she reached full feeding by the 10th-day post-operation. Also, repeated irrigations of the distal colon were done in order to dilate it.

**Figure 4:**
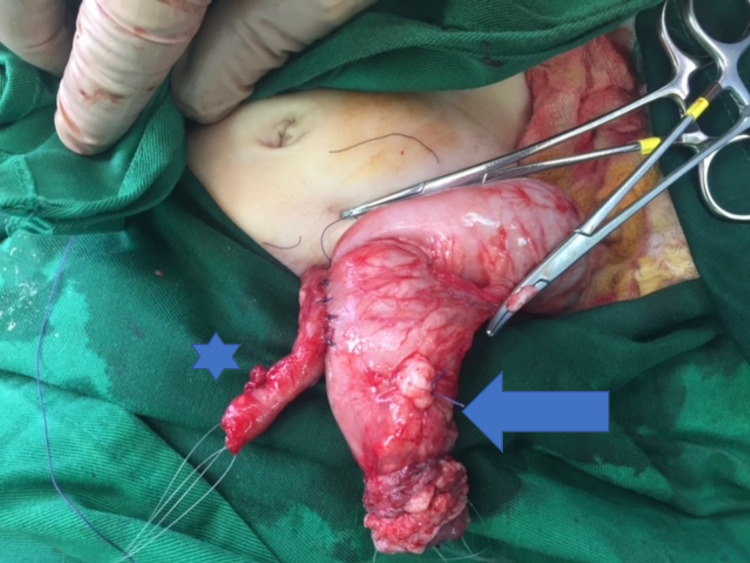
The modified Bishop-Koop procedure showing side-to-side anastomosis between the proximal and small distal open colostomies. Arrow: proximal opened colostomy, Star: distal open colostomy.

**Figure 5:**
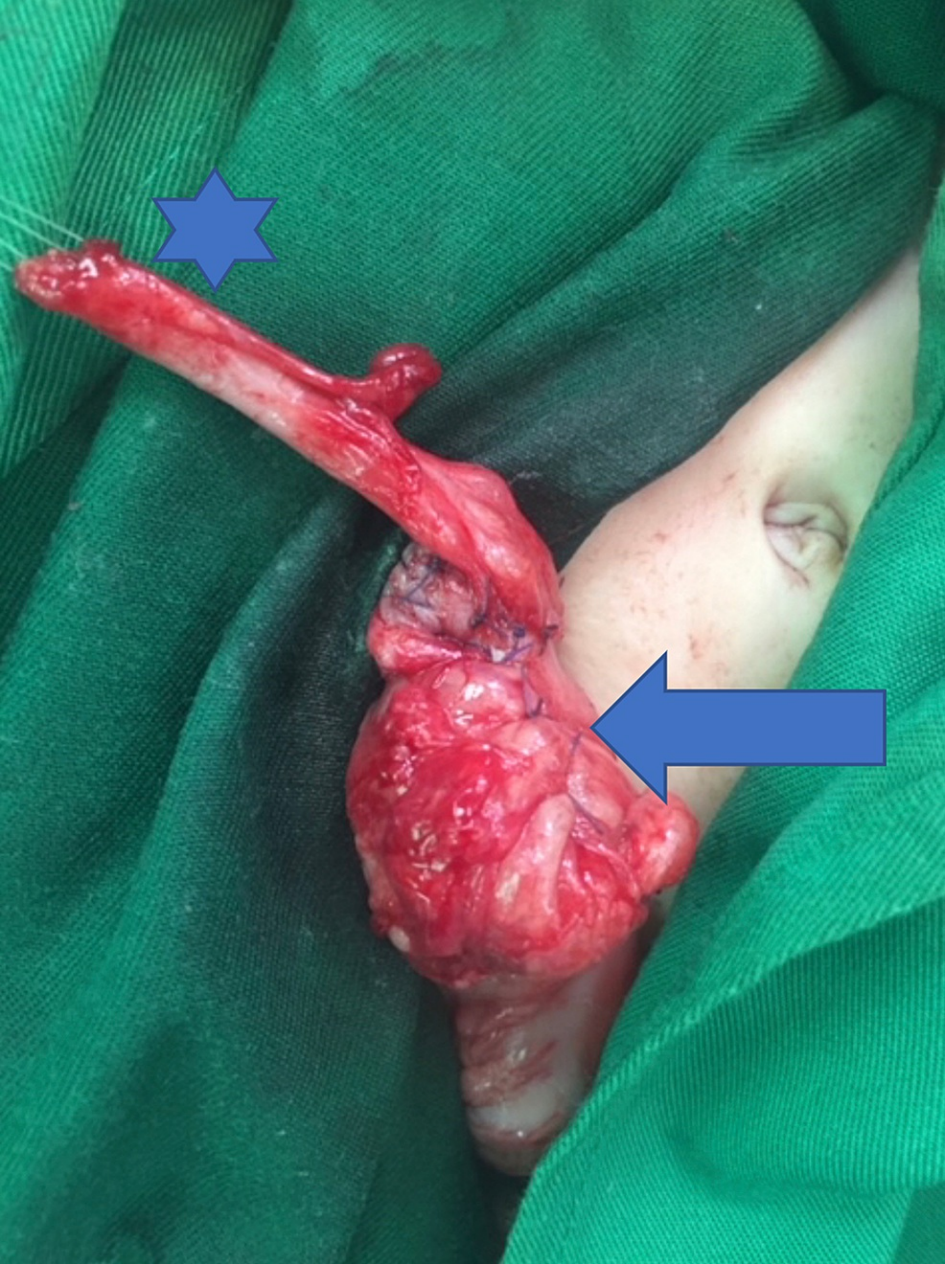
The modified Bishop-Koop procedure showing closure of the proximal end colostomy. Arrow: proximal closed colostomy, Star: distal open colostomy.

After two months, the baby was admitted to the hospital again for evaluation and possible colostomy closure. During the operation, the proximal colon diameter was still larger than the distal colon by sixfolds. Due to the discrepancy between the diameter of the two ends, the operation was converted into a distal modified chimney (Bishop-Koop) stoma. Using the side of the proximal colon as a wider end, the proximal colostomy was closed and side-to-side anastomosis was done with the distal colostomy (Figures 4, 5).

**Figure 6:**
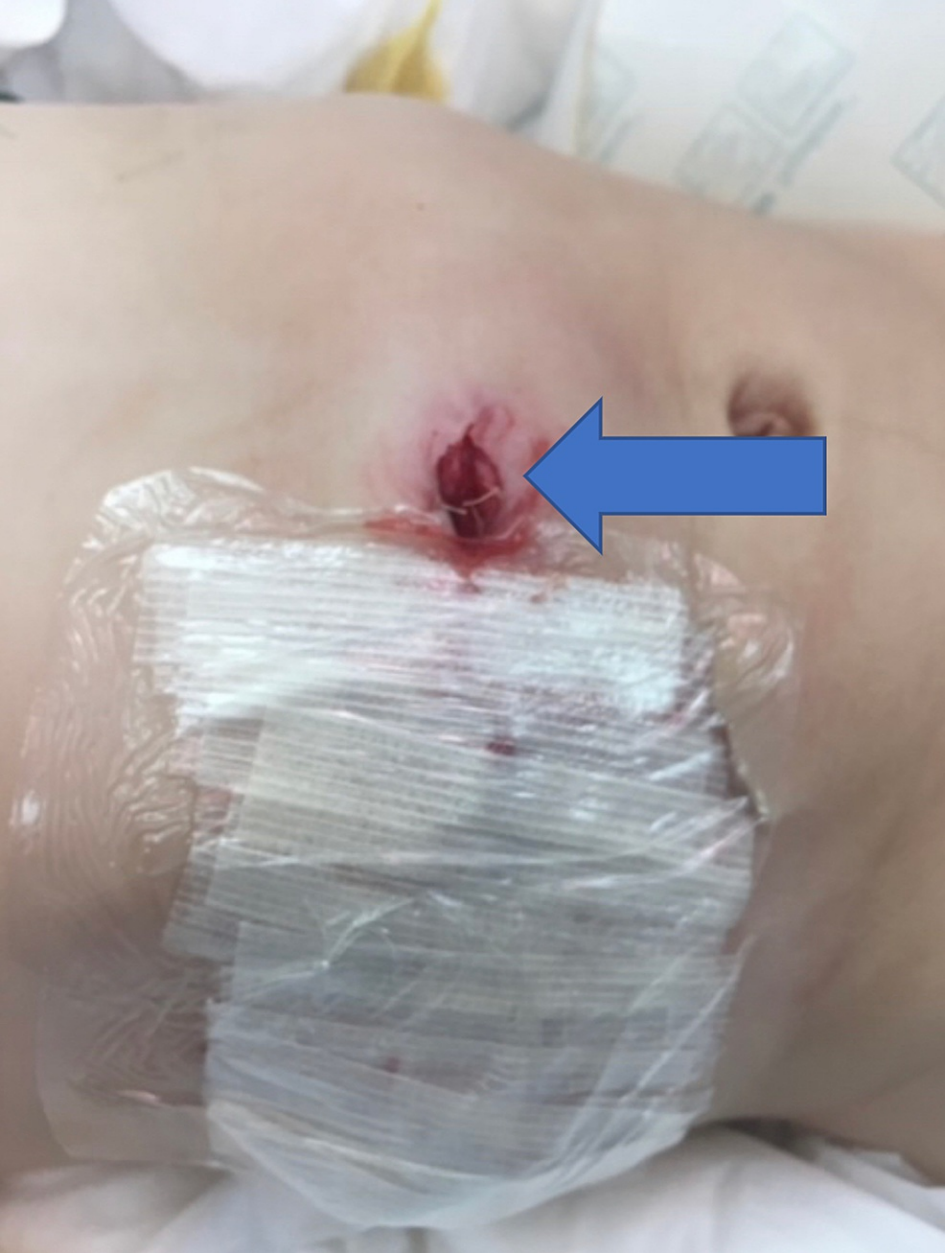
The modified Bishop-Koop procedure showing the re-exteriorized distal mucous fistula. Arrow: the re-exteriorized distal mucous fistula.

Then the distal mucous fistula was re-exteriorized (Figure 6).

After the modified Bishop-Koop procedure, there was little stool coming out from the mucous fistula. Three days after the operation, all the stool was coming out from the rectum without any passage through the mucous fistula.

Following the patient one month after the modified Bishop-Koop procedure, the patient was admitted for colostomy closure. During the operation, the caliber of the proximal and distal colon was matching. Therefore, the mucous fistula was closed successfully. After the operation, the patient was tolerating the feeding well and the wound healed completely. In addition, she passed stool normally and the abdomen was soft and lax with no complications.

## Discussion

The exact etiology behind CA development is still unknown. However, there are some theories regarding CA. According to one of them, a mesenteric vascular insult happens during fetal development [1]. Due to the latency of this vascular insult, all the features of the large colon are preserved such as taenia [1]. Volvulus, intussusception, and spontaneous thrombosis may lead to segmental ischemia and eventually atresia [5]. According to a case series done by Benawra et al. supporting the genetic factors, the cases were first-degree relatives, but there is not enough information about family history in these reported cases [6]. Multiple congenital anomalies are linked with CA accounting for 47% [2]. For instance, CA has been associated with an ocular anomaly, facial anomaly, multiple intestinal atresias, and abdominal wall defect [7]. In addition, one of the theories that explain the incidence of gastroschisis in CA patients is the compression of the bowel on the hernial or umbilical rings [7]. For treated CA patients with decreased return of gastric function, a rectal biopsy is recommended. This is because of the association between CA and Hirschsprung’s disease [8].

Focusing on CA signs and symptoms, a retrospective study done in India concluded that the common presenting signs and symptoms were distended abdomen, constipation, and bilious vomiting [9]. Moreover, meconium-stained penis and dehydration can be presented in CA patients [10]. Our patient presented with abdominal distention, bilious vomiting, jaundice, and high-grade fever. Using barium enema, obstructed microcolon and the backflow of barium are findings seen in CA patients [11]. Although the barium enema is beneficial in the neonatal period, the intraluminal pressure adjustment is crucial. This is due to potential distal colon perforation during the procedure [2]. Ultrasound (US) may reveal increased echogenicity in the distal small bowel and proximal large bowel along with dilation of the involved areas [11]. Moreover, the features of the large colon such as haustra can be detected in the US with the colon located at the margins of the abdomen [2]. In our case, an X-ray and gastrografin enema in diagnosing the patient.

Regarding the management of CA, staged surgery was preferred over primary anastomosis due to the potential risk of primary bowel anastomosis [10]. Nowadays, primary anastomosis is the procedure of choice for uncomplicated CA. In order to avoid the anastomosis complications, staged reconstruction with proximal diversion is recommended in patients with left colonic and sigmoid atresia [8,10]. Moreover, a proximal diversion was combined with primary anastomosis in half of the cases [10]. Stoma stenosis and wound dehiscence were reported as postoperative complications [10]. The Bishop-Koop procedure is defined as the creation of distal end-stoma after anastomosing the end of the proximal stoma with the side of the end stoma [12]. According to a retrospective study done in China on 41 patients with severe jejunoileal atresia, the Bishop-Koop procedure was an effective method in treating these patients with a lower mortality rate [12]. However, there were no studies discussing the usage of the Bishop-Koop procedure in treating complicated CA patients. This is due to the rarity of CA cases with the lack of a uniform guideline for managing these patients. According to our center experience in performing modified Bishop-Koop procedures, the patient underwent staged reconstruction for CA management without any complications postoperatively. Moreover, the patient underwent a modified Bishop-Koop procedure where we used side-to-side anastomosis instead of end-to-side anastomosis of the proximal and distal bowel. This is because the side-to-side anastomosis in the modified Bishop-Koop provides a larger surface area between the proximal and distal end by using the side of the proximal bowel as a wider end. As a result, a larger amount of stool will come out from the anus in a shorter period of time preventing it from irritating the skin and excoriating it. Moreover, the complete diversion of stool from the mucous fistula to the anus will happen at a shorter period in comparison to the regular Bishop-Koop procedure. 

## Conclusions

In summary, this case was about a four-day-old female patient who presented with complicated congenital CA. We have concluded that early diagnosis, by clinical and radiological means, and intervention using modified Bishop-Koop staged procedure lead to good prognosis and early recovery. Further studies comparing other surgical procedures to Bishop-Koop are recommended to improve the quality of management in CA patients.
